# Neurabin Contributes to Hippocampal Long-Term Potentiation and Contextual Fear Memory

**DOI:** 10.1371/journal.pone.0001407

**Published:** 2008-01-09

**Authors:** Long-Jun Wu, Ming Ren, Hansen Wang, Susan S. Kim, Xiaoyan Cao, Min Zhuo

**Affiliations:** Department of Physiology, University of Toronto, Toronto, Onatrio, Canada; Vrije Universiteit Amsterdam, Netherlands

## Abstract

Neurabin is a scaffolding protein that interacts with actin and protein phosphatase-1. Highly enriched in the dendritic spine, neurabin is important for spine morphogenesis and synaptic formation. However, less is known about the role of neurabin in hippocampal plasticity and its possible effect on behavioral functions. Using neurabin knockout (KO) mice, here we studied the function of neurabin in hippocampal synaptic transmission, plasticity and behavioral memory. We demonstrated that neurabin KO mice showed a deficit in contextual fear memory but not auditory fear memory. Whole-cell patch clamp recordings in the hippocampal CA1 neurons showed that long-term potentiation (LTP) was significantly reduced, whereas long-term depression (LTD) was unaltered in neurabin KO mice. Moreover, increased AMPA receptor but not NMDA receptor-mediated synaptic transmission was found in neurabin KO mice, and is accompanied by decreased phosphorylation of GluR1 at the PKA site (Ser845) but no change at the CaMKII/PKC site (Ser831). Pre-conditioning with LTD induction rescued the following LTP in neurabin KO mice, suggesting the loss of LTP may be due to the saturated synaptic transmission. Our results indicate that neurabin regulates contextual fear memory and LTP in hippocampal CA1 pyramidal neurons.

## Introduction

Synaptic plasticity, such as long-term potentiation (LTP) and long-term depression (LTD), is thought to be important for memory formation and storage in the brain [1]–[3]. Hippocampal CA1 is one of the most investigated central regions for synaptic plasticity [4]–[7]. Postsynaptic AMPA receptor trafficking, which is mainly dependent on receptor phosphorylation modulated by intracellular signaling molecules, including protein kinase and protein phosphatases, has been reported to contribute to synaptic potentiation and depression [8]–[10]. Most native AMPA receptors consist of GluR2 plus GluR1 or GluR3 subunits in CA3-CA1 synapses. At least two phosphorylation sites at GluR1 carboxyl terminals, such as protein kinase A (PKA) site and CaMKII/PKC site, are critically involved in GluR1 trafficking and bidirectional synaptic plasticity [8], [11], [12]. The phosphatases, including protein phosphatase-1 (PP1) and protein phosphatase-2B (PP2B), modulate synaptic plasticity by dephosphorylating AMPA receptors, NMDA receptors and CaMKII [13]–[16].

Mammalian neurabin (neurabin I) and spinophilin (neurabin II) are structurally similar cytoskeletal proteins, which were first isolated as F-actin-binding proteins from the rat brain [17]–[19]. In addition to actin-binding domains in N-terminal, neurabin and spinophilin contain several other domains critical for their functions, such as protein phosphatase 1 (PP1)-binding motif, PDZ domain and C-terminal coiled-coil domains [20], [21]. Neurabin and spinophilin are highly enriched in the dendritic spines of prefrontal and hippocampal neurons [22], [23], where they may serve as an adaptor protein recruiting the related binding molecules important for spine morphogenesis, synaptic transmission and plasticity.

In a previous report, reduced LTD but not LTP was found in hippocampus of spinophilin knockout (KO) mice [24]. More recently, studies have shown that neurabin is involved in the modulation of synaptic plasticity in hippocampal slice culture or neostriatal slice preparation [20], [25], [26]. Using virus-mediated expression of mutant neurabin, which is unable to bind PP1 or F-actin, it was found to attenuate LTD, increase LTP and decrease basal synaptic transmission in hippocampal slice culture [25], [26]. However, in corticostriatal synapses, Allen et al. found genetic deletion of neurabin impaired LTP while LTD was unaffected [20]. These findings indicate that the role of neurabin in central plasticity is likely region dependent. Presently, no study on hippocampus LTP and LTD has been reported in neurabin KO mice. Moreover, the role of neurabin in behavioral memory is unknown [20], [27]. Here, we investigated fear memory, basal synaptic transmission, short-term and long-term synaptic plasticity (LTP and LTD) in adult hippocampal CA1 neurons of wild-type and neurabin KO mice. Our results strongly suggest that neurabin plays a critical role in hippocampal LTP and its related contextual fear memory.

## Results

### Impaired Contextual Fear Memory in Neurabin KO Mice

**Figure 1:**
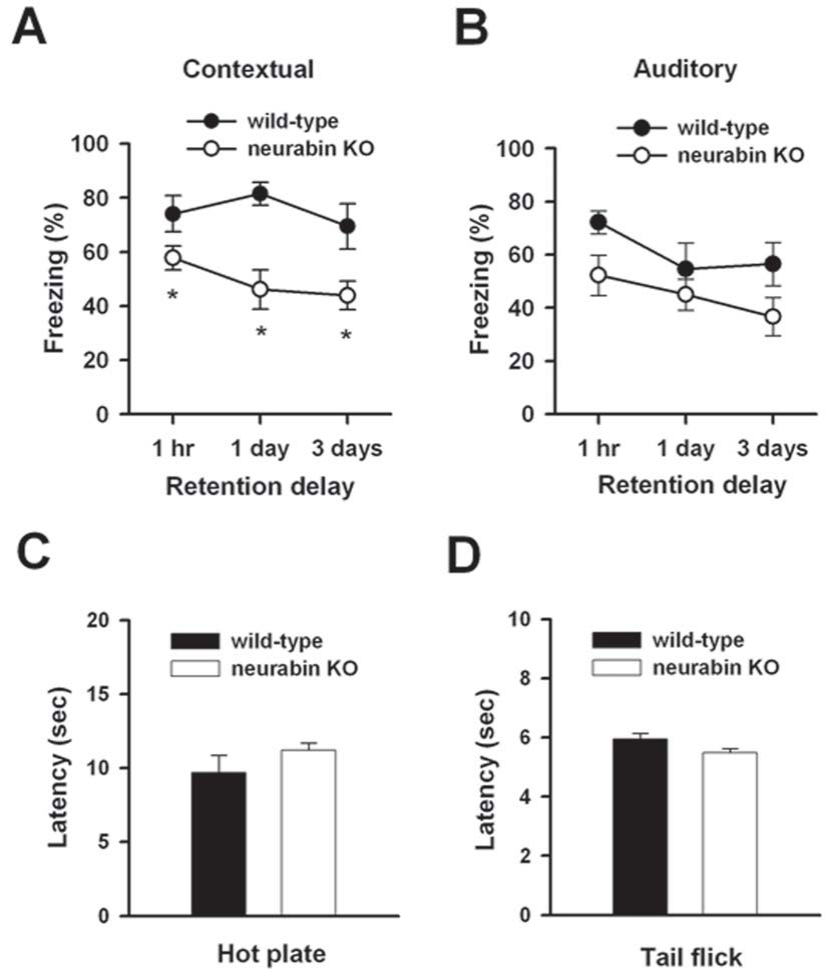
Impaired contextual but not auditory fear memory in neurabin KO mice. A, Contextual fear conditioning in neurabin KO mice (n = 6) is significantly reduced as compared with wild-type mice (n = 8) at 1 h and 1 and 3 d after training. B, Auditory fear conditioning in neurabin KO mice is similar to that in wild-type. C, There was no significant difference in response latencies between wild-type and neurabin KO mice in hotplate test. D, Spinal nociceptive tail-flick reflex between wild-type and neurabin KO mice is indistinguishable.

We assessed two forms of associative emotional memory in wild-type and neurabin KO mice: contextual and auditory fear conditioning. Contextual fear memory was first measured at 1 hour, 1 and 3 days after conditioning [28]. There was no significant difference in freezing responses immediately after training among wild-type mice (n = 8) and neurabin KO mice (n = 6), suggesting that the deletion of neurabin did not cause any developmental defect that would interfere with the shock-induced freezing response. However, neurabin KO mice showed a significant reduction in contextual fear memory one hour after conditioning (P<0.05, one-way ANOVA, Fig. 1A). The reduction in contextual fear memory lasted at one day (P<0.05) and three days (P<0.05) after conditioning (Fig. 1A). Unlike contextual fear memory, auditory fear memory was unaltered in neurabin KO mice (n = 6) compared with that of wild-type mice (n = 8; Fig. 1B).

To determine whether the reduction in contextual fear memory of neurabin KO mice is attributable to changes in pain sensitivity to the foot shock, we measured acute sensory thresholds in the hotplate and tail-flick tests. We found there is no difference between nociceptive responses of neurabin KO (n = 6) and wild-type mice (n = 8) for the hotplate set at 55°C (P>0.05, one-way ANOVA; Fig. 1C). Similarly, no difference was found in the tail-flick latency of neurabin KO mice (n = 6) compared with wild-type mice (n = 8, P>0.05, one-way ANOVA; Fig. 1D). These results indicate that the behavioral responses of neurabin KO and wild-type mice to acute noxious stimuli were indistinguishable.

### Reduced LTP but Normal LTD and Short-term Synaptic Plasticity in Neurabin KO Mice

**Figure 2:**
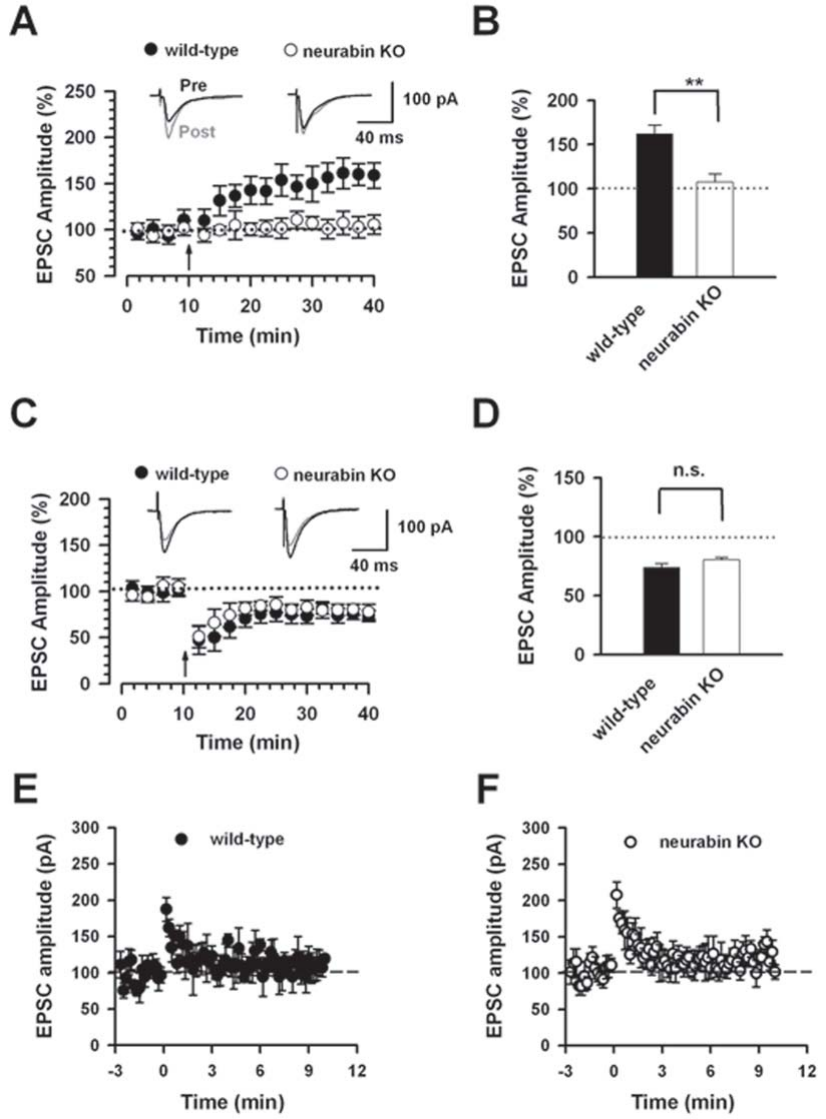
Reduction of hippocampal long-term potentiation but not long-term depression in neurabin KO mice. A, LTP is induced in hippocampal CA1 neurons with paring protocol in wild-type mice (n = 6). However, no LTP was induced in CA1 neurons in neurabin KO mice (n = 6). The insets show averages of six EPSCs at baseline response (pre, dark traces) and 30 min after (post, gray traces) LTP induction (arrow). The dashed line indicates the mean basal synaptic response. B, Statistical results showed significant loss of LTP in neurabin KO mice. C, LTD is induced in hippocampal CA1 neurons with paring protocol (−45 mV, 1 Hz, 300 pulses) in wild-type mice (n = 6). Similar LTD was observed in CA1 neurons in neurabin KO mice (n = 8). The insets show averages of six EPSCs at baseline response and 30 min after LTD induction (arrow). The dashed line indicates the mean basal synaptic response. D, Statistical results showed no significant difference (n.s.) in LTD between wild-type mice and neurabin KO mice. E, Post-tetanic potentiation is induced by stimulation of 100 Hz, 1s in wild-type mice (n = 9). F, Same stimulation protocol as E induced similar post-tetanic potentiation in neurabin KO mice (n = 12).

Hippocampal synaptic plasticity is known to be important for contextual fear memory [3], [29]. Therefore, we decided to study the role of neurabin in the synaptic plasticity, both LTP and LTD, in hippocampal CA1 pyramidal neurons. First, we investigated LTP in wild-type and neurabin KO mice. Evoked EPSCs were obtained by delivering focal electrical stimulation in striatum radium. LTP is reliably induced by the pairing protocol (see Methods) in the CA1 pyramidal neurons in wild-type mice (30 min after induction, 162.3±9.9 % of baseline response, n = 6; Fig. 2A and B). However, using the same protocol, a significantly smaller synaptic potentiation was induced in neurabin KO mice (107.6±9.4 % of baseline response, n = 6; Fig. 2A and B). To confirm the conclusion, we also studied spike-timing protocol-induced LTP (see Methods) in both groups. Similarly, there is a significant reduction of LTP in neurabin KO mice (96.6±1.4 % of baseline response, n = 6) compared to wild-type mice (135.9±10.5 % of baseline response, n = 5, P<0.01, unpaired t-test). Therefore, the deletion of neurabin abolished LTP in hippocampal CA1 pyramidal neurons.

Next, we examined LTD in wild-type and neurabin KO mice. Using pairing protocol, LTD was induced in hippocampus slices from wild-type mice (74.2±2.9 % of baseline response, n = 6; Fig. 2C and D). A similar depression was also found in neurabin KO mice by LTD induction (80.3±1.9 % of baseline response, n = 8; P>0.05 compared with wild-type mice, unpaired t-test, Fig. 2C and D). Taken together, these results suggest that neurabin contributes selectively to LTP but not LTD in the hippocampal CA1 pyramidal neurons.

Post-tetanic potentiation (PTP) is an important short-term plasticity of central synapses [30], [31]. Therefore, we measured PTP in wild-type and neurabin KO mice (Fig. 2E and F). PTP was induced by a 1 s, 100 Hz stimulus in the presence of AP5 and picrotoxin. We found that PTP exhibited no change in neurabin KO mice (187.4±16.3 % of baseline response; n = 9) compared with that in wild-type mice (207.5±18.3 % of baseline response, n = 12, P>0.05, unpaired t-test). To test whether the presynaptic release probability is altered in the KO mice, we measured responses to paired-pulse stimulation. We found that paired-pulse facilitation (PPF) is significantly increased in neurabin KO mice (n = 10) compared with wild-type mice (n = 10; P<0.05, two-way ANOVA; Fig. S1), suggesting that the release probability is decreased in neurabin KO mice.

### Normal NMDA Receptor-Mediated Synaptic Transmission in Neurabin KO Mice

**Figure 3:**
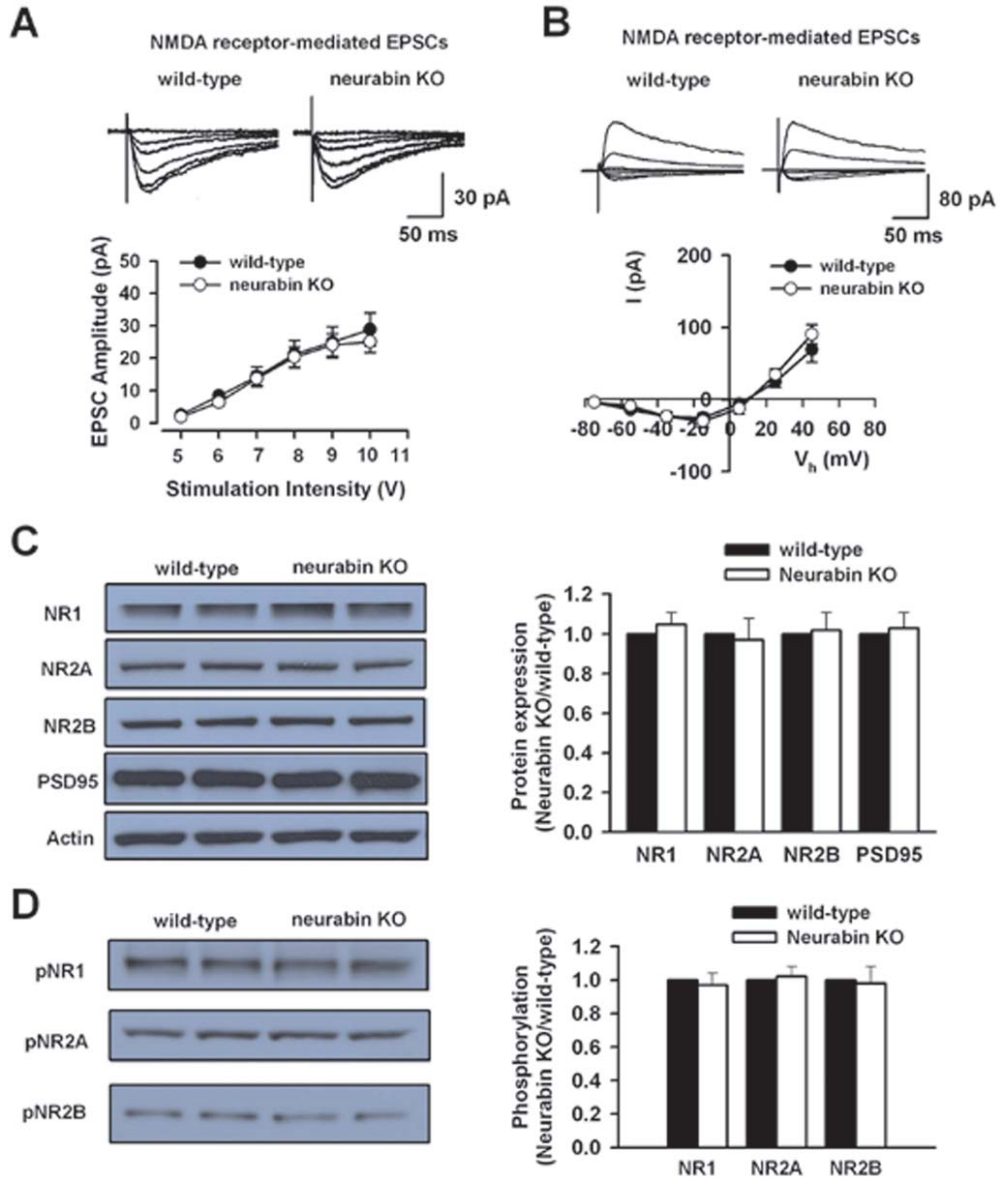
Normal function and expression of hippocampal NMDA receptor in neurabin KO mice. A, Input-output relationship for NMDA receptor-mediated EPSCs in neurabin KO (n = 8) is similar to that in wild-type mice (n = 7). B, Current-voltage plots for NMDA receptor-mediated EPSCs are not different between neurabin KO (n = 9) and wild-type mice (n = 7). C, Total expression of NMDA receptor subunits, NR1, NR2A, NR2B and PSD95 is unaltered in neurabin KO mice (n = 4 mice) compared with those in wild-type mice (n = 4 mice). D, The phosphorylation of NMDA receptor subunits, NR1, NR2A and NR2B is similar in neurabin KO mice (n = 4 mice) and in wild-type mice (n =  4 mice).

NMDA receptor is known to be important for synaptic potentiation in the CA1 region of the hippocampus. Thus, to test whether NMDA receptor function is altered or not in neurabin KO mice, we analyzed NMDA receptor-mediated EPSCs evoked by various stimulus intensities in wild-type and neurabin KO mice. We found no difference in NMDA EPSCs in neurabin KO mice (n = 8) compared with those in wild-type mice (n = 7, P>0.05, two-way ANOVA; Fig. 3A).

We also tested the voltage dependence of NMDA receptor-mediated currents. NMDA EPSCs at different holding potentials from −75 mV to +45 mV with 20 mV steps were measured and then the current-voltage (I-V) relationships of EPSCs were plotted. The results showed that I-V relationship of the NMDA-mediated EPSCs remained unchanged in neurabin KO mice (n = 9) compared with the control (n = 7; P>0.05, two-way ANOVA, Fig. 3B). These results suggest the function of NMDA receptors in neurabin KO mice is normal.

### Biochemical Studies of NMDA Receptors

We further examined the expression of different subunits of NMDA receptors, NR1, NR2A and NR2B in hippocampus of wild-type and neurabin KO mice. We found that there is no difference in the expression of NR1 (n = 4 mice, P>0.05, paired t-test), NR2A (n = 4 mice, P>0.05, paired t-test) or NR2B (n = 4 mice, P>0.05, paired t-test) between wild-type and neurabin KO mice (Fig. 3C). Moreover, we studied the expression of NMDA receptor scaffolding protein, PSD95 in neurabin KO mice. We found that PSD95 expression is not altered in neurabin KO mice (n = 4 mice, P>0.05, paired t-test, Fig. 3C).

Phosphorylation of NMDA receptor is critical for the receptor activity and PP1, a neurabin-binding protein, is known to regulate protein phosphorylation [14], [32]. Therefore, we wanted to know whether phosphorylation of NMDA receptor was altered or not in neurabin KO mice. We compared the phosphorylation of all three NMDA receptor subunits at serine sites in wild-type mice to those in neurabin KO mice. No significant change was found for the phosphorylation of NMDA receptor subunits (Fig. 3D; n = 4 mice, P>0.05, paired t-test). Taken together, these results indicate the function of NMDA receptor is intact in neurabin KO mice.

### Increased AMPA Receptor-mediated Synaptic Transmission in Neurabin KO Mice

**Figure 4:**
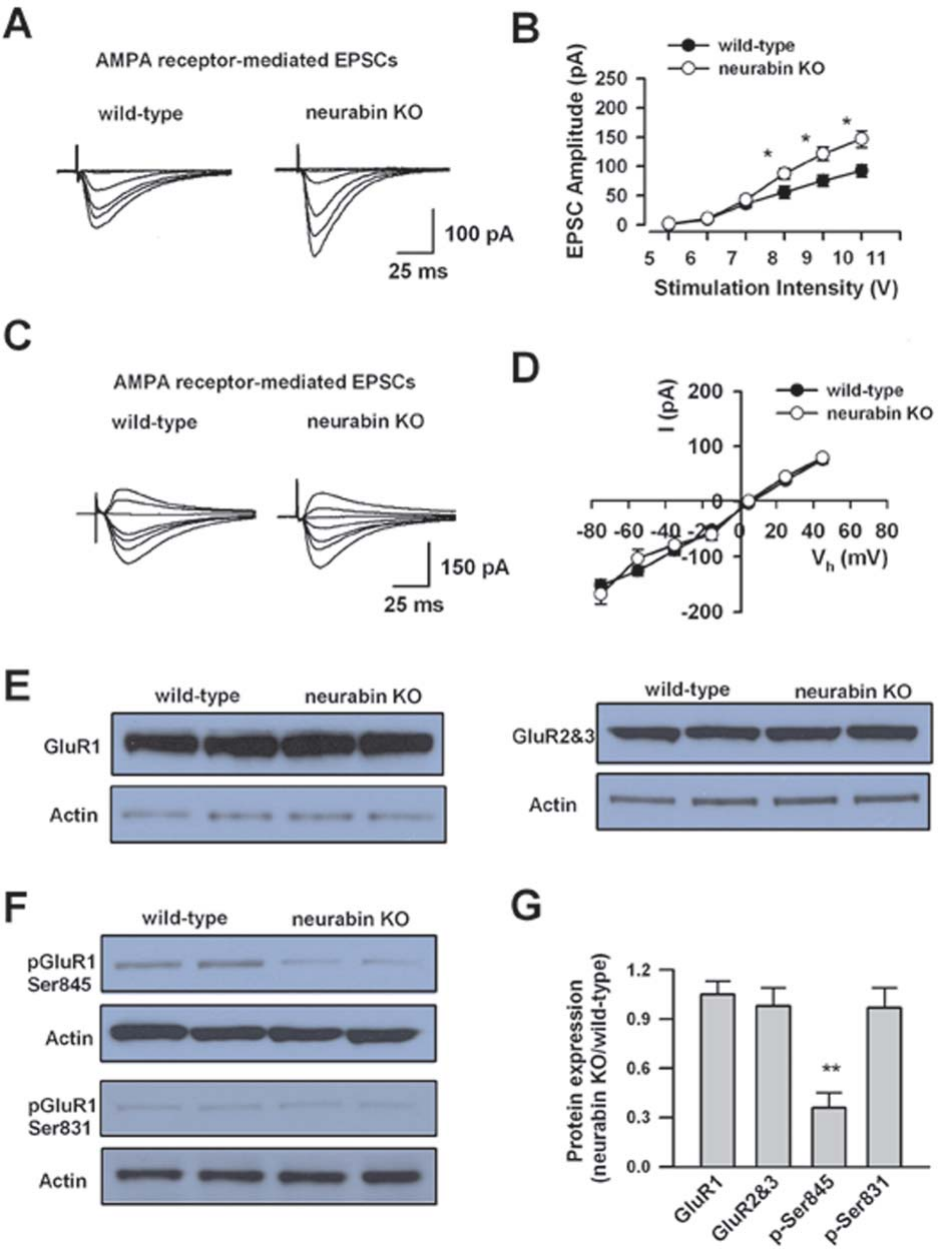
Increased hippocampal AMPA receptor-mediated EPSCs and altered GluR1 phosphorylation in neurabin KO mice. A, Representative traces of AMPA receptor-mediated EPSCs evoked by series of stimulations in wild-type and neurabin KO mice B, Input-output relationship for AMPA receptor-mediated EPSCs in neurabin KO (n = 6) and wild-type (n = 6) mice. There is a significant increase of input-output relationship for AMPA receptor-mediated EPSCs in neurabin KO mice. C and D, Current-voltage plots for AMPA receptor-mediated EPSCs in neurabin KO (n = 7) and wild-type mice (n = 7). There were no significant difference between neurabin KO mice and wild-type mice. E, Western blot showing total expression of GluR1 and GluR2&3 in neurabin KO mice (n = 4 mice) and wild-type mice (n = 4 mice). F, Western blot showing phosphorylation of GluR1 at sites of Ser845 and Ser831 in neurabin KO mice (n = 4 mice) and wild-type mice (n = 4 mice). G, Summarized results showing the relative expression of GluR1, GluR2&3, and p-Ser845 and p-Ser831 on GluR1, in neurabin KO mice (n =  4 mice) compared with those in wild-type mice (n = 4 mice). Only p-Ser845 is significantly reduced in the KO mice.

Because the neurabin-binding protein, PP1, is well known to regulate AMPA receptor activity [32], we wanted to know whether the basal synaptic transmission was altered in neurabin KO mice. To address this question, we analyzed the input-output relationship of AMPA receptor-mediated EPSCs in wild-type and neurabin KO mice. We found that AMPA receptor-mediated EPSCs in neurabin KO mice (n = 6) were significantly increased compared to those in wild-type mice (n = 6, P<0.05, two-way ANOVA; Fig. 4A and B). The enhanced EPSCs are not due to the possible change in the voltage dependence of AMPA receptor-mediated currents, since the I-V relationship of AMPA receptor-mediated EPSCs between neurabin KO mice (n = 7) and wild-type mice (n = 7; P>0.05, two-way ANOVA) is similar (Fig. 4C and D). Therefore, these results indicate that while electrophysiological properties of AMPA receptors remain unchanged, the AMPA receptor mediated responses are enhanced in neurabin KO mice.

### Biochemical Studies of AMPA Receptors

To explore the possible mechanism for the enhanced AMPA receptor-mediated responses, biochemical experiments were conducted. The increased AMPA receptor-mediated synaptic responses may reflect the altered expression or phosphorylation of AMPA receptors in neurabin KO mice [20]. To test the possibilities, we studied the expression of main AMPA receptor subunits, GluR1 and GluR2&3 in the hippocampus (Fig. 4E). We found that there is no difference in the expression of total GluR1 between wild-type (n = 4 mice) and neurabin KO mice (n = 4 mice, P>0.05, paired t-test; Fig. 4E and G). Moreover, no difference was found for total GluR2&3 expression between two groups (n = 4 mice for each group, P>0.05, paired t-test; Fig. 4E and G).

Phosphorylation and dephosphorylation of GluR1 play a critical role for AMPA receptor insertion and internalization [9], [12], thereby affecting AMPA receptor-mediated synaptic responses. We therefore studied the phosphorylation of GluR1 at two main sites, Ser845 (PKA site) and Ser831 (CaMKII/PKC site) (Fig. 4F). Surprisingly, we found that phosphorylation of PKA site was decreased in neurabin KO mice to 36.0±9.0 % of the levels in wild-type mice (n =  4 mice, P<0.01, paired t-test; Fig. 4F and G). However, phosphorylation of GluR1 at CaMKII/PKC site was not altered in neurabin KO mice (n = 4 mice, P>0.05, paired t-test; Fig. 4F and G). Therefore, deletion of neurabin regulates GluR1 phosphorylation in a site-specific manner.

### Rescue of Hippocampal LTP in Neurabin KO Mice by Pre-conditioning with LTD Induction

**Figure 5:**
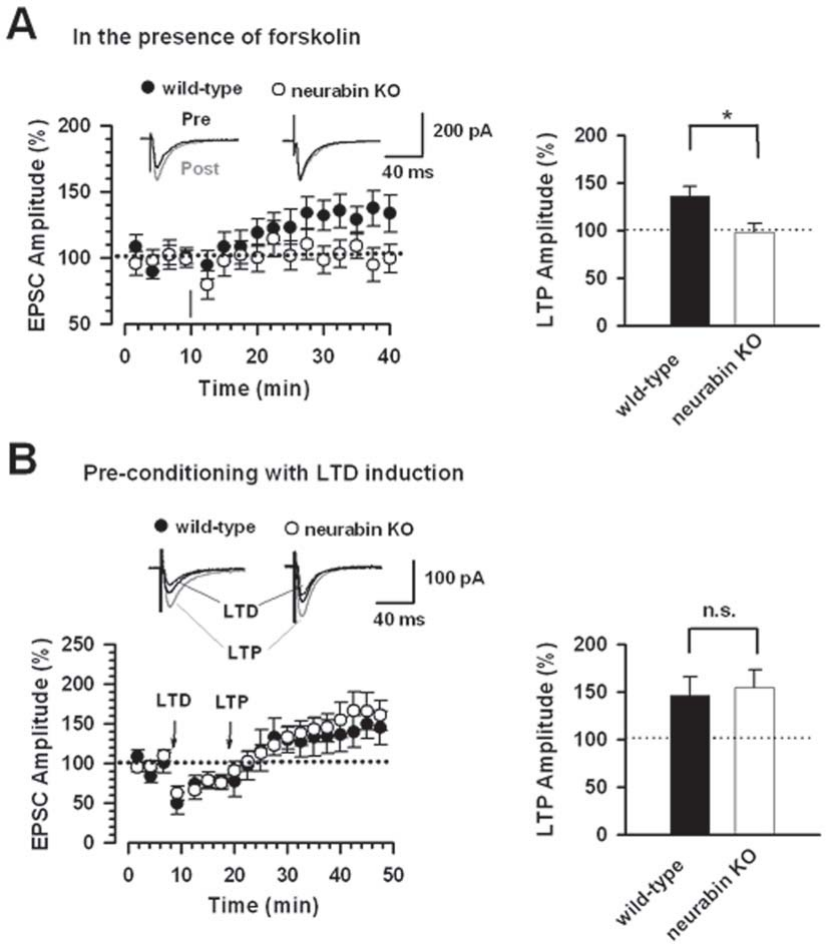
Rescue of LTP by preconditioning with LTD induction but not by forskolin treatment in neurabin KO mice. A, In the presence of forskolin (10 µM), LTP is induced in hippocampal CA1 neurons with pairing protocol in wild-type (n = 7) but not in neurabin KO mice (n = 8). The insets show averages of six EPSCs at baseline response and 30 min after LTP induction (arrow). Right panel shows the statistical result of LTP reduction in neurabin KO mice in the presence of forskolin. The dashed lines indicate the mean basal synaptic response. B, Pre-conditioning with LTD induction rescued LTP in neurabin KO mice (n = 9). The insets show averages of six EPSCs at baseline response (thick dark traces), 10 min (thin dark traces) after LTD induction (arrow) and 30 min (gray traces) after LTP induction (arrow). Right panel showing LTP in both wild-type (n = 5) and neurabin KO mice after preconditioning with LTD induction. The dashed lines indicate the mean basal synaptic response.

At least two mechanisms may underlie the loss of LTP observed in neurabin KO mice. First, the reduced phosphorylation of GluR1p845 in the neurabin KO may affect activity-dependent AMPA receptor contribution to LTP. Second, the synaptic responses may be saturated and simply could not undergo activity-dependent potentiation. To test the first idea, we examined whether forskolin, an adenylyl cyclase activator, could rescue the loss of LTP by increasing PKA activities and therefore phosphorylation of GluR1 at Ser845 site. Perfusion of forskolin (10 µM) for 10 minutes significantly increased AMPA EPSCs in hippocampal CA1 neurons in wild-type mice (180.9±20.6 % of control; n = 5, P<0.05, paired t-test) (Fig. S2). Similar increase was also observed in neurabin KO mice (168.5±21.5 % of control; n = 5, P>0.05 compared with wild-type mice, unpaired t-test). LTP induced by the pairing protocol was then studied in the presence of forskolin in both groups (Fig. 5A). We found that LTP could be observed in wild-type mice (135.9±10.0 % of baseline response, n = 7, P>0.05 compared with control LTP, unpaired t-test). However, LTP is still significantly impaired in the neurabin KO mice in the presence of forskolin (98.0±9.4 % of baseline response, n = 8, P<0.05 compared with wild-type, unpaired t-test) (Fig. 5A). Therefore, increasing cAMP-PKA activity could not rescue the loss of LTP in neurabin KO mice.

Next, we examined whether pre-conditioning with LTD induction could rescue following LTP induction in neurabin KO mice. Similar to Fig. 2C and D, LTD could be observed in both wild-type (58.3±5.0 % of baseline response, n = 5) and neurabin KO mice (68.8±5.6 % of baseline response, n = 9) 10 minutes after induction (Fig. 5B). More importantly, we found that the LTP is observed in neurabin KO mice with pre-LTD conditioning (154.6±19.1 % of baseline response, n = 9). The magnitude of potentiation is similar to that in wild-type mice (146.4±19.8 % of baseline response, n = 5, P>0.05, unpaired t-test) (Fig. 5B). Therefore, preconditioning with LTD induction could rescue the loss of LTP in the neurabin KO mice.

## Discussion

While neurabin is known for its function in actin-binding and spine morphogenesis, few studies have focused on its role in hippocampal plasticity and its related behaviors. Due to the lack of selective inhibitors for neurabin, it is impossible to study functions of neurabin in synaptic and behavioral functions using pharmacological approaches. In the present study, we found that genetic deletion of neurabin reduced contextual fear memory, but not auditory fear memory and acute sensory sensitivity. For synaptic plasticity, we found the reduced LTP, but not LTD or short-term plasticity in neurabin KO mice. These findings provide the first evidence, to our knowledge, that neurabin regulates hippocampus-related fear memory and plays different roles in hippocampal LTP/LTD as compared with spinophilin KO mice [24]. Together with the recent evidence from neostriatum [20], it suggests that neurabin may play a critical role in learning and memory in the mammalian brain.

### Neurabin Regulates Synaptic Transmission

Function of AMPA receptor is modulated by different mechanisms, including phosphorylation and their membrane expression [8], [9]. In this report, we found that AMPA receptor-mediated EPSC is significantly increased in the neurabin KO mice. At least three possibilities may account for the enhancement. First, the expression of AMPA receptor may be upregulated in neurabin KO mice. This possibility is excluded, since the total expression of GluR1 or GluR2&3 in hippocampus is similar between wild-type and neurabin KO mice. However, the results may reflect a preferential synaptic expression of AMPARs in the neurabin KO mice. Second, presynaptic glutamate release may be increased in the KO mice. However, this is not the case, as we found that PPF is increased in KO mice, suggesting the release probability may be decreased in KO mice. Third, the phosphorylation of AMPA receptor may be altered in neurabin KO mice. Indeed, our biochemical results showed that phosphorylation of GluR1 is significantly decreased at PKA site (Ser845), whereas phosphorylation is unaltered at the CaMKII site (Ser831). The results are particularly interesting, considering the both sites could be regulated by PP1 [9], [12]. One possible explanation for the results is that neurabin-binding protein PP1 may not directly target the phosphorylated sites on GluR1 subunit in dendritic spines, but through other substrates instead. For instance, PP1 could inhibit CaMKII function [15], [33], thereby affecting the phosphorylation on GluR1p831.

Recently, it has been reported that phosphorylation of GluR1p845 in the neostriatum was reduced in neurabin KO mice [20]. The amplitude of mEPSC, which reflects the function of postsynaptic AMPA receptor, however, is not altered in neostriatal slices [20]. In contrast, it was found that acute expression of neurabin deletion mutant decreases mEPSC amplitude in cultured hippocampal slice [25], [34]. The reason for the discrepancy may reside in: (1) Hippocampal cultured neurons/slices may represent functions of neurabin in early developmental stages. The present studies were performed from adult hippocampal slices. (2) Inhibition of neurabin stimulated motility and the number of dendritic spines [34]. The possibly reduced spine size may lead to the decreased responses of single synaptic event. However, the increased spine density and synapses may keep the synaptic input constant [34] or increased as shown in the present study. (3) The discrepancy of evoked EPSCs and mEPSC observed in neurabin KO mice could be reconciled since evoked response and spontaneous response may recruit different presynaptic release pools [35] as well as the postsynaptic receptor pools (Kavalali E., personal communication).

### Neurabin Regulates Synaptic Plasticity

PP1 is known to play a major role in the control of LTP and LTD in hippocampal CA1 neurons [36]. For example, the inhibition of PP1 abolished LTD and activated LTP [15], [16], [37], [38]. Considering the interaction of neurabin with PP1, it is reasonable to believe that neurabin is important in targeting PP1 to the dendritic spine, thereby modulating long-term plasticity. Indeed, it has been reported that neurabin is involved in synaptic plasticity in neostriatum and hippocampus [20], [25], [26]. Our results showed that LTP but not LTD is reduced in adult hippocampal slices from neurabin KO mice. These results suggest that modulation of neurabin on the synaptic plasticity may depend on brain regions, age of animals, slice preparations, genetic manipulations or acute versus chronic lack of neurabin.

Reduction of LTP but not LTD in the neurabin KO mice is interesting, since PP1 is critical for the induction of LTD [38] and deletion of spinophilin selectively reduced LTD but not LTP [24]. Several mechanisms may account for the altered synaptic plasticity observed in neurabin KO mice. First, since PKA and CaMKII act in parallel in controlling synaptic incorporation of GluR1 [39], reduced phosphorylation of GluR1p845 may affect activity-dependent AMPA receptor trafficking. For example, the reduction in Ser 845 phosphorylation seen in the neurabin KO may decrease the pool of “primed” receptors, and hence inhibit LTP. To test this idea, we studied the LTP in the presence of forskolin. We found that LTP is still impaired in neurabin KO mice, suggesting that decreased in “primed” pool of GluR1 is not the mechanism. Second, since AMPA receptor-mediated EPSC is enhanced in the KO mice, the synaptic responses may have already been saturated and thus affect the following LTP induction. If this is the case, unsaturating synaptic transmission with a prior induction of LTD should “rescue” LTP in neurabin KO mice. Indeed, we found LTP is observed by preconditioning the KO mice with LTD induction (de-depression), suggesting that saturated EPSCs impair the LTP induction in neurabin KO mice. However, we have to keep in mind that de-depression is a different type of synaptic plasticity, which might utilize very different induction mechanisms from that of LTP. Third, neurabin and spinophilin co-localized in spines to a large extent [22]. Therefore, the normal LTD in neurabin KO mice may be due to the functional redundancy between these two proteins and spinophilin may compensate in some way for the lost neurabin. Fourth, neurabin-binding PP1 may not directly modulate LTP or LTD. That is, some other binding proteins other than PP1 recruited by neurabin in the dendritic spine may be involved in the modulation synaptic plasticity. Further studies are clearly needed to investigate the exact signaling pathways for neurabin-related LTP.

### Neurabin Regulates Contextual Fear Memory

Despite the critical role of neurabin in synaptic formation and plasticity [20], [34], [40], much less is known about its role in behavioral functions. In the present study, we demonstrate for the first time that neurabin is involved in contextual fear conditioning, a form of associative memory [29]. Neurabin KO mice showed reduction in contextual fear memory, while auditory fear memory and acute pain thresholds were unchanged. Therefore, the impairment of contextual fear memory is unlikely due to general developmental defects or reduced sensation for foot shock. The deficit in contextual but not auditory fear memory suggests the possible alterations in hippocampus-related synaptic transmission and plasticity [29]. Consistently, we found increased AMPA receptor-mediated synaptic transmission and reduced LTP in hippocampal CA1 neurons. Since the amygdala is another brain region known to be important for contextual fear memory, future studies are needed to address the role of neurabin in synaptic functions of the amygdala. Indeed, a recent study showed an increased spinophilin containing spines in the lateral amygdala after fear conditioning [27]. It has been shown that neurabin and spinophilin play different roles in mediating behavioral responses to cocaine administration [20]. Interestingly, in contrast to what we found in neurabin KO mice, our previous studies showed that hippocampal LTD but not LTP is affected in spinophilin KO mice [24].

## Materials and Methods

### Animals

Both neurabin KO mice and genetic background matched wild-type mice were kindly provided by Dr. Paul Greengard (The Rockefeller University) and maintained in University of Toronto animal facility. Experiments were performed in mice aged between 6–14 weeks old. All mice were maintained on a 12 h light/dark cycle with food and water provided *ad libitum*. The Animal Studies Committee at the University of Toronto approved all experimental protocols.

### Fear Memory

Fear conditioning was performed in an isolated shock chamber (Med Associates, St. Albans, VT) and performed in a blind manner to the genotypes of mice. The conditioned stimulus (CS) was an 85 dB sound at 2800 Hz, and the unconditioned stimulus (US) was a continuous scrambled footshock at 0.75 mA. After 2 min of habituation, animals received the CS/US pairing [a 30 s tone (CS) and a 2 s shock (US) starting at 28 s; three shock–tone pairings were delivered at 30 s intervals], and the mice remained in the chamber for an additional 30 s to measure immediate freezing. At 1 hour, 1 and 3 days after training, each mouse was placed back into the shock chamber and the freezing response was recorded for 3 min (contextual conditioning). Subsequently, the mice were placed into a novel chamber and monitored for 3 min before the onset of a tone identical to the CS, which was delivered for 3 min, and freezing responses were recorded (auditory conditioning).

### Hot Plate Test and Tail-flick Reflex

In the hotplate test, mice were placed on a standard thermal hotplate with a 10*10 inch black heated surface at 55°C (Columbus Instruments, Columbus, OH). The latency in which the mice showed signs of nociception, by either rapid fanning or licking of the hindpaws, was recorded. A cutoff time of 30 s was used to prevent injury. The spinal nociceptive tail-flick reflex was evoked by radiant heat (Columbus Instruments) applied to the underside of the tail. The latency to reflexive removal of the tail away from the heat was measured by a photocell timer. A cutoff time of 10 s was used to minimize the skin damage.

### Whole-cell Patch Clamp Recordings

Coronal brain slices (300 µm) containing the hippocampus from six- to eight-week-old wild-type and neurabin KO mice were prepared using standard methods [41]. Slices were transferred to a submerged recovery chamber with oxygenated (95 % O_2_ and 5 % CO_2_) artificial cerebrospinal fluid (ACSF) containing (in mM: 124 NaCl, 2.5 KCl, 2 CaCl_2_, 2 MgSO_4_, 25 NaHCO_3_, 1 NaH_2_PO_4_, 10 glucose) at room temperature for at least 1 h.

Experiments were performed in a recording chamber on the stage of an Olympus BX51WI microscope (Tokyo, Japan) with infrared DIC optics for visualization of whole-cell patch clamp recordings [42]. Excitatory postsynaptic currents (EPSCs) were recorded from hippocampal CA1 pyramidal neurons with an Axon 200B amplifier (Molecular devices, CA) and the stimulations were delivered by a bipolar tungsten stimulating electrode placed in striatum radium. The recording pipettes (3–5 MΩ) were filled with solution containing (mM): 145 CsMeSO_3_, 5 NaCl, 1 MgCl_2_, 0.2 EGTA, 10 HEPES, 2 Mg-ATP, 0.1 Na_3_-GTP, 5 QX-314 (adjusted to pH 7.2 with CsOH). When using spike-time protocol for LTP induction, CsMeSO_3 _was replaced by equimolar K-gluconate and QX-314 chloride was omitted in the internal solution. For induction of LTP with pairing protocol, cells were held at +30 mV while Schaffer collateral afferents were stimulated at 2 Hz for 80 pulses. The spike-timing protocol for LTP involved paired 3 presynaptic stimuli which caused 3 EPSPs (10 ms ahead) with 3 postsynaptic action potentials at 30 Hz, paired 15 times every 5 s. For induction of LTD with pairing protocol, cells were held at −45 mV while Schaffer collateral afferents were stimulated at 1 Hz for 300 pulses. To compare the I-V curve of NMDA or AMPA EPSCs, similar EPSC amplitudes were induced in both groups by adjusting the stimulation intensity. When short-term plasticity was studied, AP5 (50 µM) was perfused in the ACSF to avoid the NMDA receptor-dependent long-term plasticity. Access resistance was 15–30 MΩ and was monitored throughout the experiment. Data were discarded if access resistance changed more than 15% during an experiment.

### Western Blot and Immunoprecipitation

The hippocampus tissues were dissected and homogenized in lysis buffer (10 mM Tris-HCl, pH 7.4 , 2 mM EDTA, 1% SDS) containing 1X protease inhibitor cocktail (Sigma, MO), and 1X phosphatase inhibitor cocktail 1 and 2 (Sigma, MO). Protein was quantified by Bradford assay. Electrophoresis of equal amounts of total protein was performed on SDS-polyacrylamide gels. Separated proteins were transferred to polyvinylidene fluoride membranes at 4°C for Western blot analysis. Membranes were probed with 1∶3000 dilution of anti-GluR1 (Upstate, NY), anti-GluR2&3 (Chemicon, CA), 1∶2000 dilution of anti-PSD95 (Chemicon, CA), 1∶1000 dilution of anti-phospho-GluR1 Ser845 (Upstate, NY) or anti-phospho-GluR1 Ser831 (Upstate, NY),and 1∶1000 dilution of anti- NR1 (Upstate, NY) anti-NR2A (Chemicon), or anti-NR2B (Chemicon) antibodies. The membranes were incubated in the appropriate horseradish peroxidase-coupled secondary antibody diluted 1∶3000 for 1 h followed by enhanced chemiluminescence (ECL) detection of the proteins with Western lightning chemiliminescence reagent plus (Perkin Elmer Life sciences, MA) according to the manufacturer's instructions. To verify equal loading, membranes were also probed with 1∶3000 dilution of anti-actin antibody (Sigma, MO). The density of immunoblots was measured using NIH Image software.

### Data Analysis and Statistics

Results were analyzed by t-test, paired t-test, or two-way ANOVA followed by post-hoc Student-Newman-Keuls test to identify significant differences. All data are expressed as mean±S.E.M. In all cases, P<0.05 was considered statistically significant.

## Supporting Information

Figure S1 Reduced glutamate release probability in neurabin KO mice A, Representative traces showing the paired-pulse facilitation (PPF) of EPSCs at time interval of 35 ms, 75 ms, 100 ms and 150 ms in the wild-type and neurabin KO mice. B, Statistical results showed that significant increase of PPF in neurabin KO mice (n = 10) compared with that of wild-type mice (n = 10).
(4.05 MB TIF)

Figure S2 Forskolin enhanced EPSCs in neurabin KO mice. A, Perfusion of forskolin (10 µM) gradually increased the amplitude of EPSCs in both wild-type (n = 5) and neurabin KO mice (n = 5). The insets show averages of six EPSCs at baseline response (dark traces) and 10 min (grey traces) after forskolin perfusion. The dashed lines indicate the mean basal synaptic response. B, Statistical results showing no difference in the increase of EPSCs by forskolin between wild-type and neurabin KO mice.
(4.20 MB TIF)
