# Supplementary figures and images for: Neurabin Contributes to Hippocampal Long-Term Potentiation and Contextual Fear Memory
Source: PLoS One. 2008 Jan 9;3(1):e1407. doi: 10.1371/journal.pone.0001407 (PMC2169299; doi:10.1371/journal.pone.0001407)

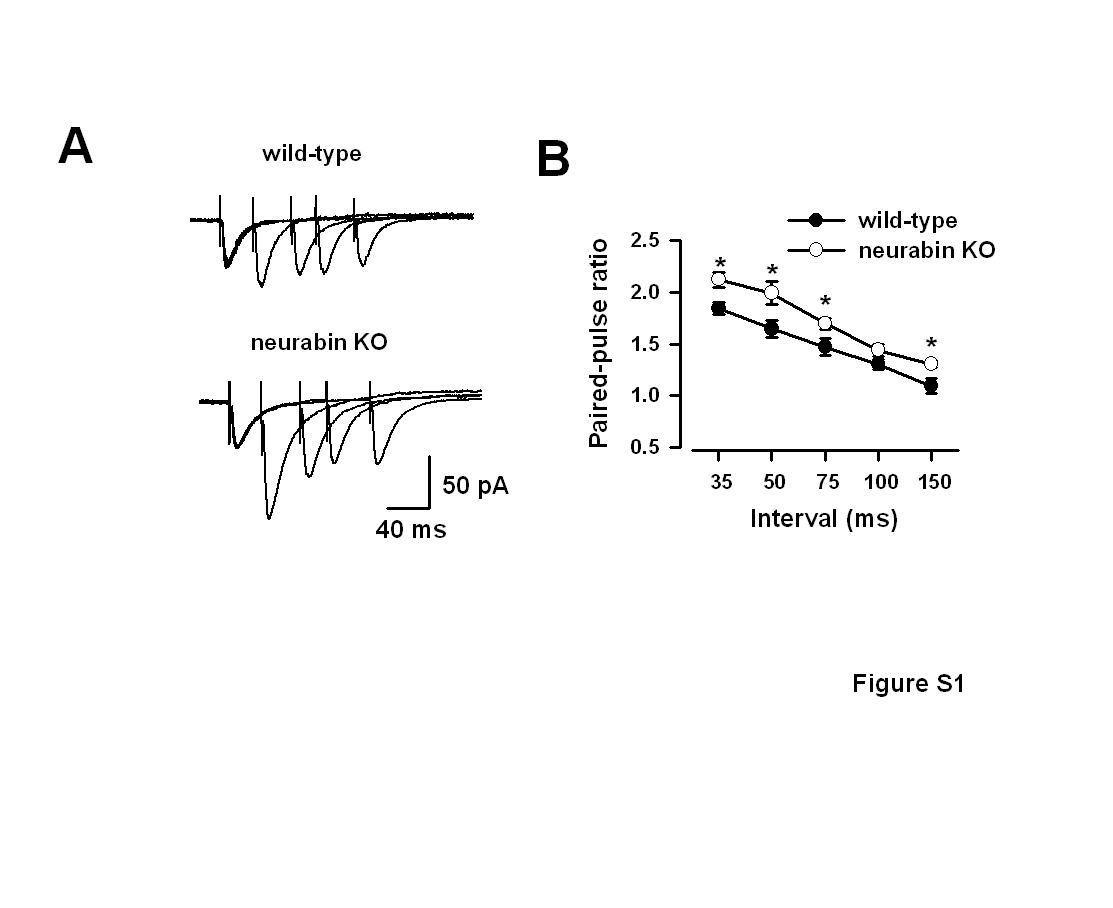

Supplement: Figure S1 — Reduced glutamate release probability in neurabin KO mice A, Representative traces showing the paired-pulse facilitation (PPF) of EPSCs at time interval of 35 ms, 75 ms, 100 ms and 150 ms in the wild-type and neurabin KO mice. B, Statistical results showed that significant increase of PPF in neurabin KO mice (n = 10) compared with that of wild-type mice (n = 10). (4.05 MB TIF) [file pone.0001407.s001.tif]

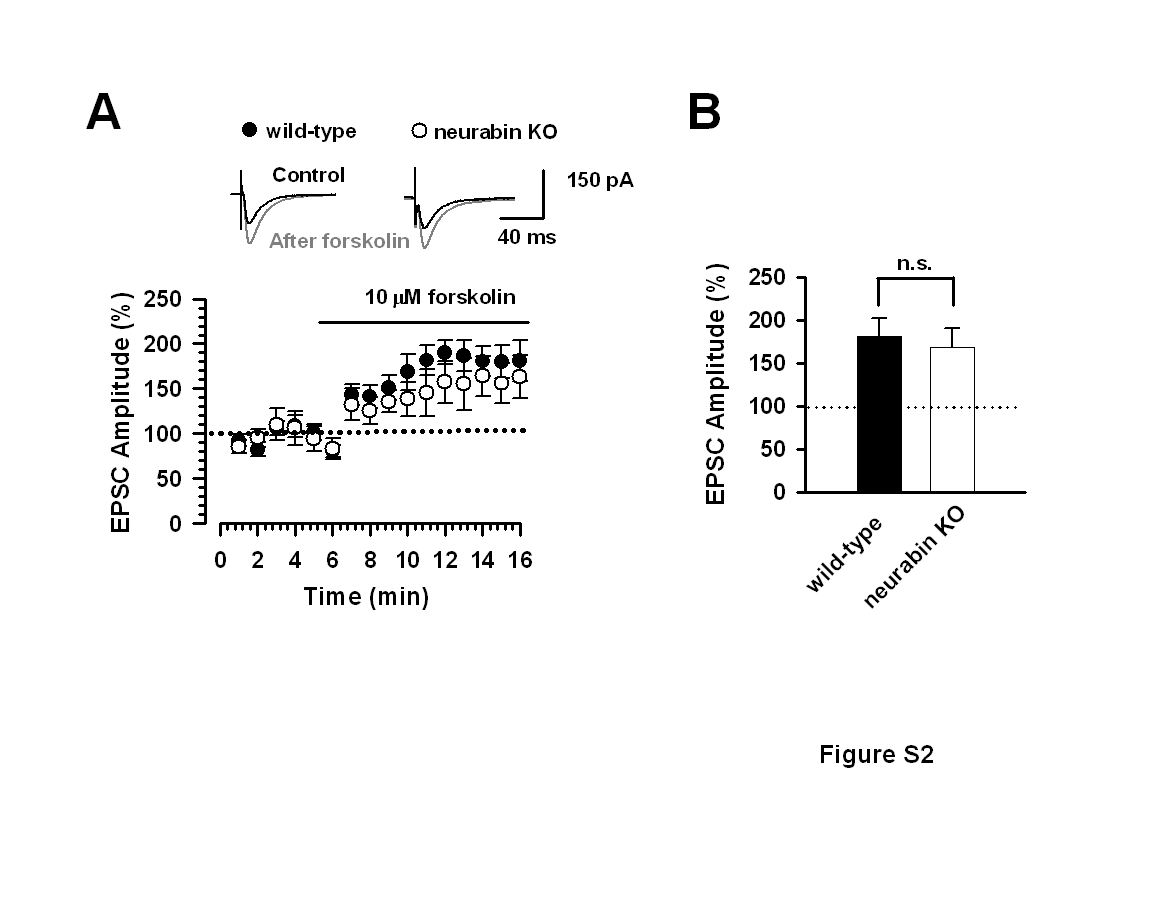

Supplement: Figure S2 — Forskolin enhanced EPSCs in neurabin KO mice. A, Perfusion of forskolin (10 µM) gradually increased the amplitude of EPSCs in both wild-type (n = 5) and neurabin KO mice (n = 5). The insets show averages of six EPSCs at baseline response (dark traces) and 10 min (grey traces) after forskolin perfusion. The dashed lines indicate the mean basal synaptic response. B, Statistical results showing no difference in the increase of EPSCs by forskolin between wild-type and neurabin KO mice. (4.20 MB TIF) [file pone.0001407.s002.tif]
